# Magnetic Resonance Imaging in Cervical Spine Trauma: More Than Soft Tissue Illustration

**DOI:** 10.7759/cureus.21493

**Published:** 2022-01-22

**Authors:** George Fotakopoulos, Alexandros G Brotis, Konstantinos N Fountas

**Affiliations:** 1 Neurosurgery, General University Hospital of Larisa, Larisa, GRC

**Keywords:** computed tomography, misdiagnosis, magnetic resonance imaging, trauma, cervical-spine

## Abstract

The role of magnetic resonance imaging (MRI) in cervical spine trauma is limited to visualizing soft tissues such as the intervertebral disc, the spinal cord, and hematomas. Herein, we present the case of a 60-year-old man who was transferred to our hospital with neck pain after a cervical spine trauma associated with a motor vehicle accident. The initial computed tomography imaging of the cervical spine showed stable linear fractures at the C2, C6, and C7 vertebral bodies, for which the patient received conservative management. The patient showed remarkable clinical improvement three months later, but the linear fractures at the subaxial spine remained unchanged on computed tomography (CT). Magnetic resonance imaging (MRI) scantly differentiated active from inactive bone lesions and prevented unnecessary interventions. Therefore, we suggest that the MRI is of value in cases with a clinical and radiological mismatch. A mismatch is considered in cases when there is a high level of clinical suspicion for a spinal fracture, whereas CT images fail to provide direct evidence of a bone fracture. In such cases, MRI offers indirect evidence of bony trauma, such as bone marrow edema, visualized as a high-intensity signal in T2-weighted images. Furthermore, specialized spine trauma MRI protocols could be of value in selected cases.

## Introduction

Initial management and diagnosis of polytrauma patients are provided in the emergency department (ED). Cervical spine fractures may add to these patients' unnecessary morbidity and mortality if not properly evaluated and treated. It is of paramount importance to correctly diagnose and classify any cervical spine fractures and ensure the appropriate treatment for every patient.

Computed tomography (CT) has become the first choice as a routine screening tool in the emergency department with a reported sensitivity as high as 98%, offering a clear advantage over other diagnostic tools in terms of the speed and convenience of the assessment. CT rarely misses spinal fractures [1]. On the other hand, magnetic resonance imaging (MRI) is the appropriate examination tool to explain the patient’s neurological status. It could show soft tissue injuries of paraspinal structures, cervical discs, neural structures, and hematomas [2].

The NEXUS (National Emergency X-Radiography Utilization Study) criteria and the C-spine rule provide algorithms for imaging the cervical spine after spine trauma using plain radiographs. To the best of our knowledge, there is no tool to guide CT and MRI in spine trauma. The aim of our study was twofold. We hypothesize that an MRI is essential in cervical spine injuries with a clinical and radiological mismatch. Second, we stress the importance of a specialized “trauma” protocol for such cases, aiming to remove potential artifacts.

## Case presentation

A 60-year-old man was transferred to our emergency department after a reported motor vehicle accident. The patient suffered severe pain in the cervical region (VAS neck 7), occipital headache, and arm numbness. There were no motor deficits in any key muscle nor any signs of myelopathy.

**Figure 1:**
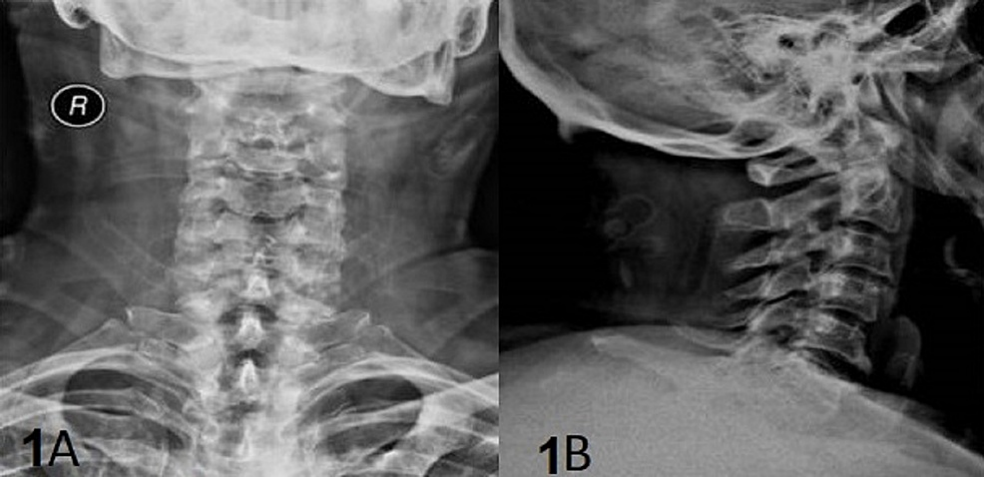
X-rays of the cervical spine A 60-year-old man patient with cervical spine's trauma who presented with severe pain of the cervical spine, headache, and numbness of his arms: (A) X-rays of the cervical spine (anteroposterior view) didn't show any pathology; (B) X-rays of the cervical spine (lateral view) didn't show any pathology. Both X-rays are performed with an orthopedic collar.

**Figure 2:**
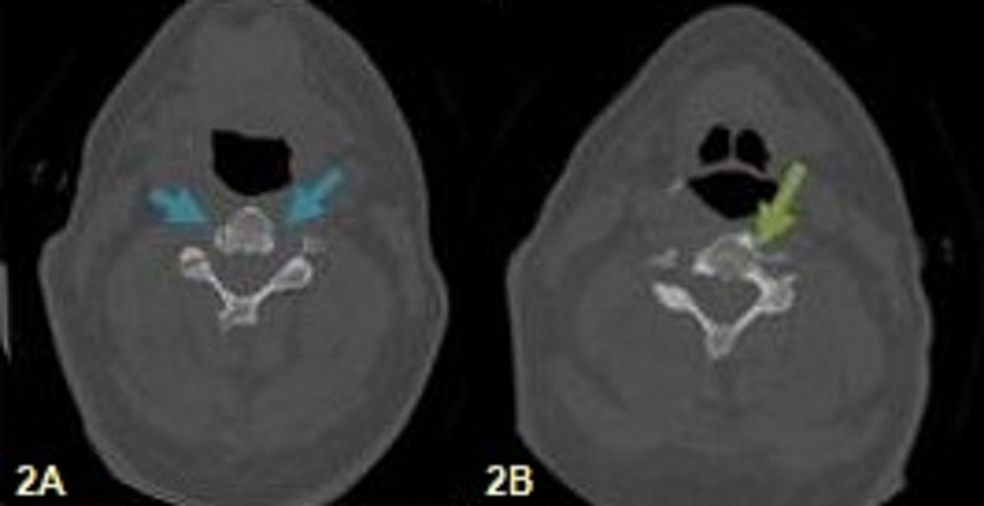
Computed tomography (CT) A 60-year-old man patient with cervical spine trauma who presented with severe pain of the cervical spine, headache, and numbness of his arms: (A) computed tomography (CT) consecutive images of C6 vertebra show the complex of blood vessels like a vertebra's fracture (blue arrows) and (B) CT consecutive images of the C7 vertebra show the blood vessels' abnormality with the course over the left neck of C7 that imitates a fracture (green arrows).

The cervical spine alignment was normal in the plain radiographs (Figure 1). The cervical spine CT showed a stable C2 vertebral body fracture. In addition, there was suspicion of a hairline fracture at the vertebral body of the C6 and C7 vertebrae (Figure 2). Accordingly, we decided on conservative management using a Miami-J cervical brace, muscle relaxants, and non-steroidal anti-inflammatory drugs (NSAIDS).

**Figure 3:**
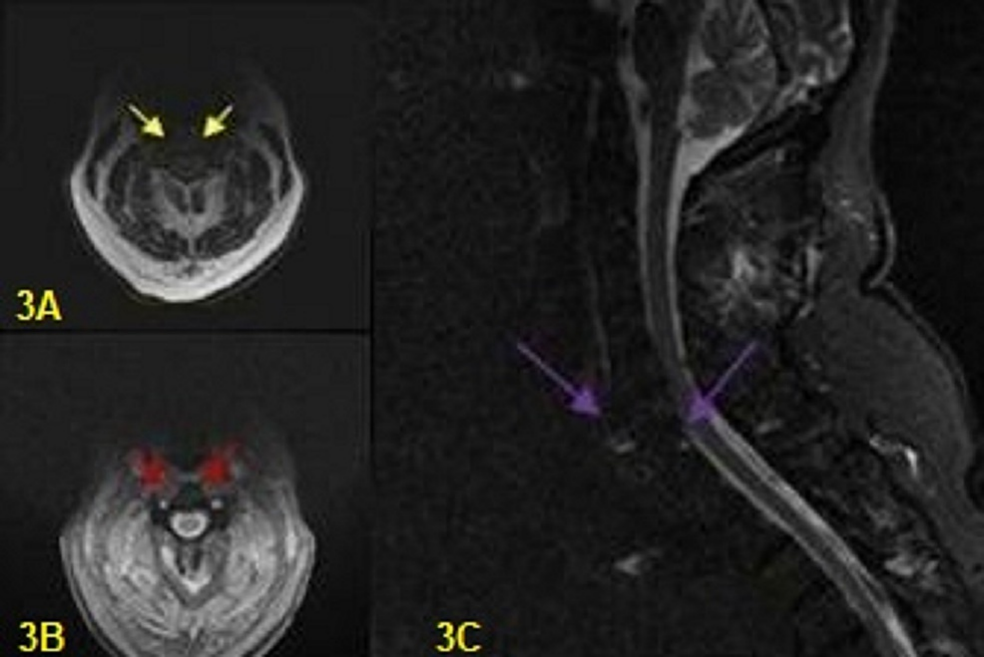
Magnetic resonance imaging (MRI) A 60-year-old man patient with cervical spine trauma who presented with severe pain of the cervical spine, headache, and numbness of his arms: (A) Magnetic resonance imaging (MRI) axial images (2D MERGE - Multiple Echo Recombined Gradient Echo) present linear enhancement signal intensity in C6 and C7 vertebras (yellow arrows); (B) Magnetic resonance imaging (MRI) axial images (3D COSMIC - Coherent Oscillatory State Acquisition for the Manipulation of Imaging Contrast) present linear enhancement signal intensity in C6 and C7 vertebras (red arrows); (C) Magnetic resonance imaging (MRI) sagittal STIR (Short Inversion Recovery) images present high signal intensity in the region of the suspicious fracture at the C6-C7 level (purple arrows)

As the symptoms persisted (visual analog scale (VAS) neck 8), the patient underwent magnetic resonance (MR) imaging of the cervical spine, which showed bone marrow edema at the C2 vertebral body, a linear hypodensity in T1 weighted images at the C6 and C7 vertebrae, and a normal spinal cord (Figure 3).

Three months later, the MRI showed less edema at C2. However, the persistence of the C6 and C7 findings with an absence of high signal intensity on T2-weighted images opted for a non-traumatic lesion, including a vascular structure. Since the patient was free of symptoms, we decided to substitute the rigid Miami-J with a soft collar for a month.

## Discussion

With the current manuscript, we try to pinpoint that radiological imaging examinations are not a panacea. The patient’s clinical presentation and the physician’s clinical examination are of utmost importance. Therefore, a negative CT scan does not necessarily exclude a minor spinal fracture, particularly in the presence of a high level of clinical suspicion. These patients should be either referred to dedicated high-volume centers or treated as potential fractures with bracing, analgesics, and regular follow-up. Given that MRI is not present in every facility all the time and it is a costly examination, a spinal MRI could be of value in selected cases with persisting symptoms or in deciding the return to work.

Upper cervical injuries consist of a diverse group of injuries that affect the skull base, atlas, and axis and often result from high-energy injuries like sudden moves of the spine and strong blows to the head [1].

CT is increasingly indicated as the sole primary screening tool in high-energy injuries, almost eliminating the need for traditional radiographs to clarify bony cervical spine injuries [1]. A mismatch is considered in cases when there is a high level of clinical suspicion for a spinal fracture, whereas CT images fail to provide direct evidence of a bone fracture. In such cases, the MRI offers indirect evidence of bony trauma, such as bone marrow edema, visualized as a high-intensity signal in T2-weighted images.

Despite the wide use of CT nowadays, sometimes, the CT images could present false-positive results that often lead to mismanagement. This misleading information may be due to motion artifacts, anatomical abnormalities, normal variations, or technique misfires [3]. Some studies presented artifacts due to motion and vascular variants that could imitate cervical spine fractures, and thus, many patients underwent unnecessary treatments [3]. Mehta et al. reported that a movement artifact in the patient's original CT scans could be misinterpreted as a unilateral facet fracture subluxation at C5-C6 vertebrae [4].

Furthermore, Coats et al. studied a case that highlights a situation in which a CT scan error led to an unnecessary patient transferring to another healthcare facility and a second CT scan [1]. The authors concluded that clinical re-evaluation is necessary whenever there is a clinical and radiological mismatch. The greatest of MRI in spinal trauma was the assessment of the soft tissue component of injury [2]. According to Finkenzeller et al., in the cervical spine, BLADE sequences MRI appears to significantly reduce motion, truncation, and flow artifacts and improve image quality to avoid misdiagnosis [5]. In our case, the MRI helped pinpoint the active bone lesion and protected the patient from unnecessary interventions.

Additionally, sagittal cervical spine imaging was shown as an improvement of the spinal cord/cerebrospinal fluid (CSF) contrast in a BLADE sequences MRI, diminishing of the CSF-flow artifacts, as well as an improvement of the depiction of surrounding structures in sagittal orientation [6]. When considering lesion recognition, the diagnostic consistency of spinal cord depiction and the reduction of artifacts are of supreme clinical significance.

At least, in the cervical region, T2-BLADE in axial orientation led to a blurring of the subarachnoid space due to superimposed CSF flow effects, which thus demotes results in the assessment of this area as compared to Turbo Spin Echo (TSE). However, for spinal cord interpretation, axial BLADE was better than TSE but without importance in the cervical spine [7].

## Conclusions

The highlights of our study were: 1) An anatomic abnormally in a cervical-spine study could appear as a traumatic fracture/subluxation and lead to a misdiagnosis; 2) It is important to exclude artifacts as a cause of normal variants and abnormal blood vessels of the cervical spine; 3) Radiologists and neurosurgeons have to be trained and be careful in relation to a cervical spine study because there are many normal variants, anatomical abnormalities, and structures that very often imitate pathologies.

To conclude, there are rare cases in which fractures of cervical vertebras could be misdiagnosed. According to the bibliography, there is a possibility to confuse a vertebral fracture with motion artifacts, abnormal fistulas, or even normal variations of blood vessels like in our patient. CT is generally indicated in cervical spine trauma to visualize bone lesions, whereas MRI is reserved for imaging soft tissue lesions. Nevertheless, MRI may frequently differentiate an active fracture from a dormant variant. Therefore, MRI is indicated in cases with a clinical and radiological mismatch.
